# Genome-Wide Identification and Expression Profiling of Brassinosteroid-Responsive WRKY Transcription Factors Potentially Involved in Natural Rubber Biosynthesis in *Hevea brasiliensis*

**DOI:** 10.3390/biology15211965

**Published:** 2026-11-01

**Authors:** Xinyu Ni, Songle Fan, Guojiao Lin, Bingbing Guo, Mingyang Liu, Lifeng Wang

**Affiliations:** 1 State Key Laboratory of Tropical Crop Breeding, Key Laboratory of Biology and Genetic Resources of Rubber Tree, Ministry of Agriculture and Rural Affairs/State Key Laboratory Incubation Base for Cultivation and Physiology of Tropical Crops, Rubber Research Institute, Chinese Academy of Tropical Agricultural Sciences, Haikou 571101, China; nixinyu_n@163.com (X.N.); slfan@catas.cn (S.F.); guobingbing1989@126.com (B.G.); liumy2023@126.com (M.L.); 2 College of Tropical Crops, Yunnan Agricultural University, Kunming 650201, China; 15184822632@163.com

**Keywords:** *Hevea brasiliensis*, WRKY transcription factor, brassinosteroid, G-box, natural rubber biosynthesis

## Abstract

Natural rubber is synthesized in the laticifers of *Hevea brasiliensis*, but the transcriptional networks regulating latex-associated processes remain poorly understood. In particular, whether WRKY transcription factors participate in brassinosteroid (BR)-responsive regulation remains unclear. We identified the HbWRKY family using the haplotype A telomere-to-telomere reference genome of *Hevea brasiliensis* and integrated phylogeny, gene structure, duplication type, synteny, *Ka*/*Ks*, promoter cis-elements, tissue expression, and BR-treated transcriptomic data. The 109 HbWRKY proteins comprised 21 Group I, 72 Group II, and 14 Group III members, together with one atypical protein and one Group I-like protein. Whole-genome or segmental duplication accounted for 102 genes (93.6%), and *Ka*/*Ks* was below 1 for all 111 duplicated HbWRKY pairs, consistent with purifying selection. G-box elements occurred in 84 promoters (77.1%). Eighty-nine genes were expressed in bark and 37 in latex, with latex showing the highest value for nine genes. Seven *HbWRKY* genes were differentially expressed after BR treatment, and all were downregulated. *HbWRKY7* (*HbAchr10M0003572*) and *HbWRKY28* (*HbAchr15M0012484*) were prioritized because both combined differential expression following BR treatment, their highest recorded expression in bark among the seven tissues, and predicted promoter G-boxes. This updated family resource narrows the candidates for testing a BR-WRKY regulatory module in rubber tree.

## Simple Summary

Natural rubber is produced mainly from the latex of the rubber tree, but the transcriptional pathways that connect plant hormones with latex-associated metabolism remain poorly defined. We catalogued WRKY transcription factors in the haplotype A telomere-to-telomere reference genome and combined evolutionary analyses with expression data from seven tissues, transcript profiles after brassinosteroid treatment, and promoter G-box predictions. The analysis identified 109 WRKY genes. Most were retained after whole-genome or segmental duplication, and their duplicated coding sequences were predominantly constrained by purifying selection. Eighty-nine genes were expressed in bark and 37 in latex. Seven WRKY genes were differentially expressed following brassinosteroid treatment, all with lower transcript abundance than in the control. Two genes, *HbWRKY7* and *HbWRKY28*, were prioritized because they were differentially expressed following brassinosteroid treatment, had their highest recorded transcript abundance in bark among the sampled tissues, and contained predicted promoter G-box elements. These convergent observations identify testable candidates for studying hormone-responsive regulation in rubber tree bark and latex-associated biology.

## 1. Introduction

Natural rubber (NR), composed mainly of cis-1,4-polyisoprene, combines elasticity, abrasion resistance, and low-temperature performance that cannot be fully reproduced by synthetic alternatives. Although several plant species synthesize rubber, the rubber tree (*Hevea brasiliensis*) remains the principal commercial source [1,2]. NR is synthesized in laticifers within the secondary phloem and is stored on rubber particles in latex. After tapping, laticifers must reallocate carbon, energy, protein synthesis, and transport capacity to regenerate latex, so yield depends on both the state of the laticifer system and the efficiency of rubber resynthesis [3,4]. Isopentenyl diphosphate, supplied mainly by the cytosolic mevalonate pathway in rubber tree latex, is polymerized from allylic diphosphate initiators at the rubber-particle surface. Cis-prenyltransferases (CPT/HRT), CPT-like proteins, rubber elongation factor, small rubber particle protein, and the HRT1-REF bridging protein contribute to assembly of the rubber biosynthetic machinery [5,6,7]. Genomic and transcriptomic studies further show coordinated changes in rubber synthesis, precursor metabolism, energy supply, hormone signaling, and protein homeostasis in laticifers [3,8,9]. The upstream transcriptional network that controls the timing and tissue specificity of these processes remains less well resolved than the enzymatic machinery itself.

Brassinosteroids (BRs) initiate signaling through BRASSINOSTEROID INSENSITIVE 1 and its co-receptor BAK1. This pathway inhibits the GSK3-like kinase BIN2 and promotes dephosphorylation and nuclear accumulation of BES1/BZR1 transcription factors, thereby altering genes involved in growth, vascular differentiation, and stress responses [10,11]. At the transcriptional level, BES1 and BZR1 regulate downstream gene expression by recognizing specific cis-regulatory elements, including BR-responsive elements (BRREs) and E-box-related motifs such as the G-box, often in cooperation with other transcription factors [12]. BR signaling also intersects with auxin, gibberellin, abscisic acid, jasmonate, and ethylene pathways, and its transcriptional output depends on tissue and environmental context. Rubber tree BRI1 and BAK1 family members have been identified, and several respond transcriptionally to exogenous hormones [13]. HbBIN2 also shows protein kinase and transcriptional regulatory activities [14]. Rubber tree therefore retains core BR perception and signaling components, but their downstream transcriptional effects in laticifers and latex metabolism remain unclear.

WRKY proteins are plant-specific transcription factors with diverse regulatory functions. A typical WRKY protein contains an approximately 60-amino-acid WRKY domain with an N-terminal WRKYGQK core and a C-terminal C2H2- or C2HC-type zinc-finger motif. The family is commonly divided into Groups I, II, and III according to WRKY-domain number and zinc-finger type, with Group II further separated into subgroups IIa–IIe [15,16]. WRKY proteins usually recognize W-box elements and can operate with VQ proteins, mitogen-activated protein kinases, and other regulators. Family members have been implicated in development, senescence, primary and secondary metabolism, biotic and abiotic stress, and hormone responses [17,18]. WRKY proteins also intersect with BR signaling. In *Arabidopsis thaliana*, WRKY46, WRKY54, and WRKY70 contribute to BR-regulated growth responses; WRKY54 interacts with BES1 and is regulated through BIN2-mediated phosphorylation and protein stability [19]. Rice OsWRKY53 participates in BR signaling and plant architecture [20]. These findings indicate that WRKY proteins can form part of BR-responsive transcriptional networks.

An earlier rubber tree genome analysis identified 81 HbWRKY genes, several of which responded to jasmonate and ethylene; HbWRKY3, HbWRKY14, and HbWRKY55 bound and activated the *HbSRPP* promoter in yeast [21]. Latex-enriched HbWRKY27 was subsequently shown to bind the *HbFPS1* promoter and increase its transcriptional activity [22]. These studies support WRKY proteins as plausible regulators of genes associated with rubber biosynthesis, but the available family inventory was based on an earlier genome version and emphasized tissue, jasmonate, and ethylene responses. Re-identification in the haplotype A telomere-to-telomere reference genome, combined with analyses of duplication history, evolutionary constraint, tissue expression, BR responsiveness, and promoter composition, can provide a more rigorous basis for candidate selection.

Here, we conducted a genome-wide analysis of the HbWRKY family using the haplotype A telomere-to-telomere reference genome. We examined protein features, phylogenetic relationships, gene structure, conserved motifs, duplication type, intra- and interspecific synteny, and selection pressure. Tissue-expression profiles, BR-treated transcriptomic data, and promoter G-box distributions were then integrated to prioritize BR-responsive HbWRKY genes with bark- or latex-associated expression. The study was designed to generate testable candidates rather than to establish direct regulation of NR biosynthesis.

## 2. Materials and Methods

### 2.1. Genome Data Collection and Genome-Wide Identification of HbWRKY Genes

The haplotype A telomere-to-telomere reference genome and gene annotation of *H. brasiliensis* cultivar CATAS73397 were used as the rubber tree reference. The assembly was identified as version 1 (Figshare DOI: 10.6084/m9.figshare.26644396.v1; NGDC BioProject PRJCA022207) (accessed on 17 June 2025) [9]. One representative protein sequence was analyzed for each retained gene model. Classified *A. thaliana* WRKY proteins were used as homology-search queries and as references for family classification.

Candidate proteins were identified by combining a profile hidden Markov model search based on Pfam entry PF03106 with an independent BLASTP search using AtWRKY proteins as queries [23]. The candidate sets were merged and duplicate protein identifiers were removed. Retained proteins were re-examined with NCBI Batch Conserved Domain Search (https://www.ncbi.nlm.nih.gov/Structure/bwrpsb/bwrpsb.cgi) (accessed on 20 June 2025). Proteins were classified by WRKY-domain number, WRKYGQK-core variation, zinc-finger type, and phylogenetic placement. The conserved domains of candidate HbWRKY proteins were further examined using SMART to assess the presence and organization of WRKY domains. Particular attention was given to proteins with atypical domain structures or variations in conserved WRKY motifs. Original CATAS73397 gene identifiers were retained to ensure traceability across analyses and avoid confusion with previously named HbWRKY genes.

### 2.2. Protein Properties, Gene Structure, and Conserved Motifs

Protein length, predicted molecular weight, and theoretical isoelectric point were obtained with ExPASy-ProtParam (https://web.expasy.org/protparam/) (accessed on 25 June 2025). Exon, intron, coding-sequence, and untranslated-region coordinates were retrieved from the CATAS73397 annotation and matched to validated HbWRKY identifiers. Gene structures and conserved-domain positions were visualized with TBtools-II 2025 (https://github.com/CJ-Chen/TBtools-II) (accessed on 27 June 2025) [24]. Subcellular localization was predicted using Plant-mPLoc and is summarized in Table S2. Conserved protein motifs were identified with MEME Suite v5.0.5 (https://meme-suite.org/meme/) (accessed on 30 June 2025) [25] using the zero-or-one-occurrence-per-sequence model, a maximum of six motifs, motif widths of 6–50 amino acids, and an E-value threshold of 10.0.

### 2.3. Phylogenetic Analysis and Classification of HbWRKY Proteins

Full-length HbWRKY and AtWRKY protein sequences were aligned with MUSCLE (https://www.ebi.ac.uk/jdispatcher/msa/muscle) (accessed on 1 July 2025) [26]. Maximum-likelihood phylogenetic inference was performed with IQ-TREE 2.4.0 (https://iqtree.github.io/) (accessed on 10 July 2025) [27]. ModelFinder (https://iqtree.github.io/ModelFinder/) (accessed on 15 July 2025) [28] selected JTTDCMut+I+G4 according to the Bayesian information criterion, and branch support was assessed with 5000 ultrafast bootstrap replicates. The tree was formatted with FigTree v1.4.4 (https://tree.bio.ed.ac.uk/software/figtree/) (accessed on 20 July 2025). HbWRKY proteins were classified into Group I, Group II (IIa–IIe), and Group III based on their phylogenetic relationships with previously classified AtWRKY proteins, together with WRKY domain number and zinc-finger characteristics. In particular, the classification of Group IIa–IIe was primarily determined by phylogenetic clustering with the corresponding *Arabidopsis* WRKY reference proteins. *Arabidopsis* proteins in nearby phylogenetic positions were linked to the literature-curated functional information summarized in Table S5.

### 2.4. Chromosomal Distribution, Gene Duplication, and Collinearity Analysis

Chromosomal coordinates were extracted from the CATAS73397 annotation and mapped to the 18 assembled chromosomes. Gene duplication and collinearity were analyzed with MCScanX (https://plantgenome.uga.edu/mcscanx-app/) (accessed on 25 July 2025) [29] using genome annotations and protein-similarity results. Genes were assigned to the duplication categories retained in the project output: whole-genome or segmental, dispersed, proximal, and singleton.

Interspecific collinearity was examined between *H. brasiliensis* and *A. thaliana* and between *H. brasiliensis* and *Manihot esculenta*. The cassava files were labeled as *M. esculenta* v8. Chromosomal and synteny plots were generated with TBtools [24]. *Manihot esculenta* was selected for interspecific collinearity analysis because it belongs to the same family (Euphorbiaceae) as *H. brasiliensis*, allowing the assessment of WRKY gene synteny between relatively closely related species. *Arabidopsis thaliana* (Brassicaceae) was included as a more distantly related reference to evaluate the conservation of WRKY gene synteny across different evolutionary distances.

### 2.5. Ka/Ks Analysis and Evolutionary Selection

Within-genome *HbWRKY* duplicate pairs were obtained from the rubber tree duplication analysis, and rubber tree-cassava WRKY pairs were obtained from the interspecific collinearity analysis. Corresponding coding sequences were retrieved from the archived rubber tree and cassava CDS collections. *Ka*, *Ks*, and *Ka*/*Ks* were calculated with Simple *Ka*/*Ks* Calculator [24]. Rows with undefined *Ks* and *Ka*/*Ks* for which the archived output reported high sequence divergence (pS ≥ 0.75) were excluded from quantitative summaries. *Ka*/*Ks* < 1, =1, and >1 were interpreted as patterns consistent with purifying selection, neutral evolution, and positive selection, respectively [30]. No branch, branch-site, or site model was applied.

### 2.6. Transcriptome Data Collection and Analysis

A previously generated expression matrix was used for bark, female flower, latex, leaf, male flower, root, and seed. The underlying public datasets were reported under SRA accessions SRR3136158, SRR3136165, SRR3136162, SRR3136159, SRR3136166, SRR3136156, and SRR3136168. Target-gene expression values across the seven tissues were extracted using the previously described method [31]. BR-treated latex transcriptomic data were generated by the authors’ laboratory and have not yet been published. A 5 μmol/L brassinosteroid (BR) solution was applied to the tapping cuts with a brush, using the concentration reported by Guo et al. (2023) [32]. The control group (CK) received water applied to the tapping cuts using the same procedure. Latex samples were collected 12 h after treatment. Three RNA-seq libraries were analyzed for each condition. Differential expression between BR and CK was analyzed with DESeq2 [33], using |log_2_ fold change| ≥ 1 and an adjusted *p* value < 0.01 as the criteria for identifying differentially expressed genes. For visualization, FPKM values were averaged across the three libraries per condition. Here, BR-responsive denotes differential expression at the 12 h sampling point and does not establish an early or direct BR response. The expression matrix of the 109 *HbWRKY* genes under CK and BR treatment conditions is provided in Supplementary Table S7.

### 2.7. Promoter Extraction and Cis-Regulatory Element Analysis

For each *HbWRKY* gene, a 2000 bp sequence upstream of the annotated 5′ gene boundary was used operationally as the promoter. Promoter sequences were submitted to PlantCARE (https://bio.tools/plantcare (accessed on 16 September 2026)). PlantCARE records labeled G-box or G-Box were normalized to G-box. All sequence variants associated with those labels were retained rather than restricting the analysis to the canonical CACGTG hexamer. Coordinates were reported in a common 5′-to-3′ promoter orientation. Repeated annotations with identical gene, start, and end coordinates were collapsed before site counts were calculated, and one or more retained intervals were scored as G-box occurrence.

### 2.8. Integrative Prioritization of BR-Responsive Candidates

Candidate genes were prioritized by intersecting BR-responsive differential expression, the seven-tissue expression profile with emphasis on bark and latex, and promoter G-box occurrence. For each BR-responsive *HbWRKY* gene, bark and latex values, the tissue with the highest expression, and the number of unique promoter G-box intervals were retrieved. Among the genes differentially expressed following BR treatment, those exhibiting their highest recorded transcript abundance in bark and containing at least one predicted promoter G-box were designated as core bark-associated candidates. Other BR-responsive genes were retained as supporting candidates according to detectable bark or latex expression and G-box occurrence. This was a descriptive, rule-based integration; no weighted score, machine-learning procedure, or additional significance test was applied.

## 3. Results

### 3.1. Genome-Wide Identification and Structural Characterization of the HbWRKY Family

**Figure 1:**
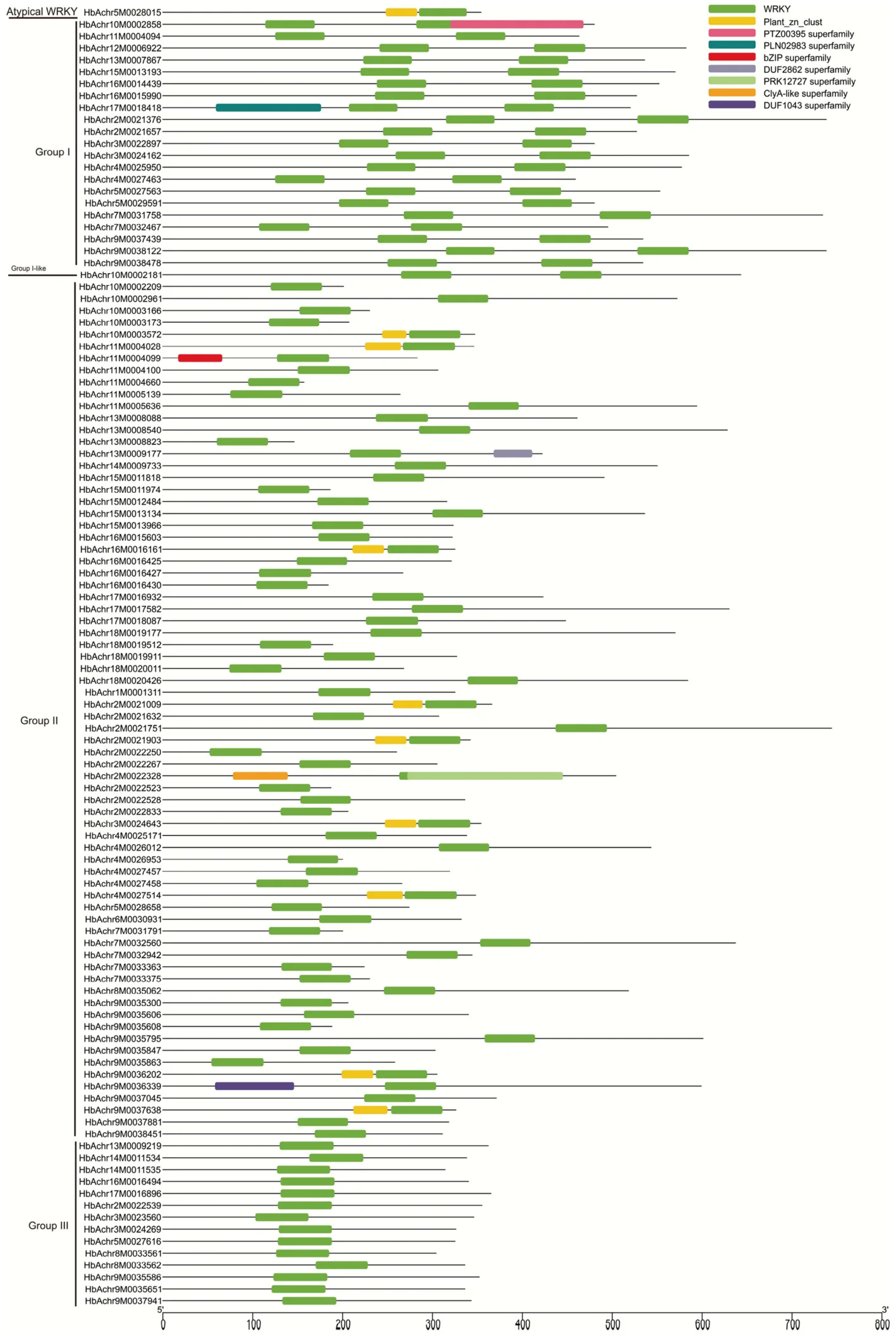
Conserved domain architecture of HbWRKY proteins in *Hevea brasiliensis*. Horizontal lines represent proteins, and colored boxes indicate predicted conserved domains and superfamilies. Proteins are arranged as atypical WRKY, Group I, Group I-like, Group II, and Group III. The WRKY domain is shown in green.

A total of 109 nonredundant HbWRKY proteins were identified in *Hevea brasiliensis*. Based on WRKY-domain number and zinc-finger characteristics, the proteins were initially assigned to different WRKY groups (Table S1). These preliminary assignments were subsequently evaluated and refined according to their phylogenetic relationships with *Arabidopsis* WRKY proteins. The resulting classification included one atypical WRKY protein, 21 Group I proteins, 72 Group II proteins, 14 Group III proteins, and one Group I-like protein (Figure 1 and Figure 3; Table S1). The proteins ranged from 145 to 743 amino acids, with a median of 343 amino acids. Predicted molecular weights ranged from 16.83 to 82.19 kDa and theoretical isoelectric points from 4.80 to 9.84 (Table S2). All HbWRKY proteins were predicted to localize to the nucleus (Table S2).

All 21 Group I proteins contained two detected WRKY domains, whereas each of the other 88 proteins contained one. Most proteins retained the canonical WRKYGQK heptapeptide: 82 single-domain proteins contained one copy, and all 21 Group I proteins contained two. Six proteins carried variant cores. HbAchr10M0003173, HbAchr11M0004660, HbAchr4M0026953, and HbAchr7M0033363 contained WRKYGKK; HbAchr16M0016427 contained WHKYGQK; and the Group III protein HbAchr3M0023560 contained WRKYGEK. Group I and Group II proteins predominantly displayed C2H2-type zinc fingers, whereas Group III proteins had the expected C2HC configuration. The atypical protein HbAchr5M0028015 retained WRKYGQK but lacked a recognizable canonical C2H2 or C2HC pattern downstream. HbAchr10M0002181 contained one intact WRKY-C2H2 domain and a second highly degenerate WRKY-like region and was designated Group I-like.

The *HbWRKY* genes exhibited variations in exon–intron organization and genomic length, with *HbAchr2M0021751* showing a particularly extended genomic span (Figure S1). Phylogenetically related genes often had comparable exon–intron organizations. MEME recovered six conserved motifs (Figure S2). Group I proteins generally showed a duplicated architecture, with an N-terminal motif 3/5 region and a C-terminal block containing motifs 6, 1, 4, 2, and 5. Most Group II proteins contained one motif 1–4–2–5 block, often preceded by motif 6, whereas Group III proteins usually retained the shorter motif 1–4–2 organization. Gene structure, motif composition, and domain architecture were therefore broadly concordant with family classification.

### 3.2. Phylogenetic Relationships, Duplication, Collinearity, and Selection

**Figure 2:**
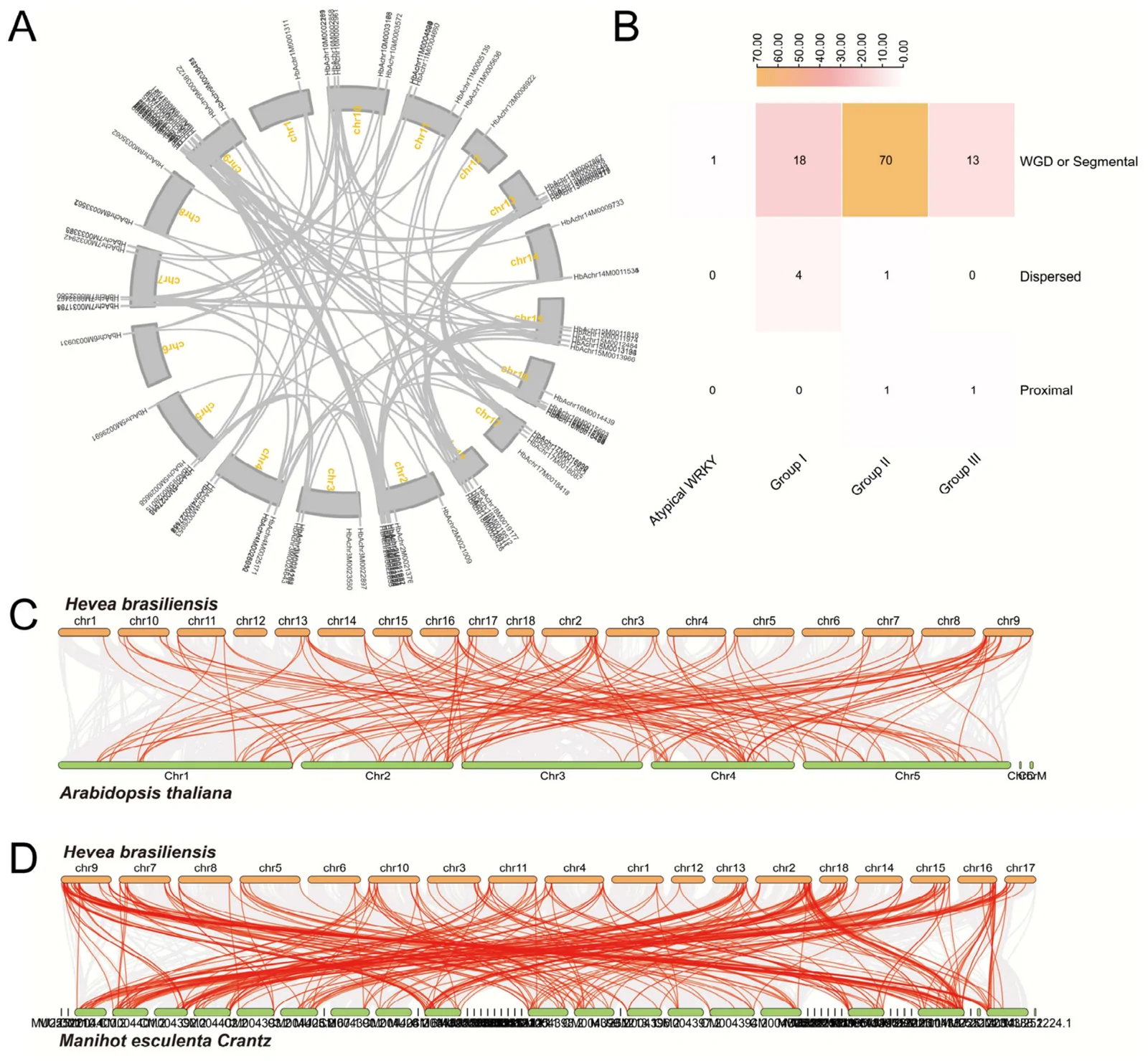
Gene duplication and comparative synteny of the *HbWRKY* family. (**A**) Chromosomal distribution and duplication links among *HbWRKY* genes; gray curves connect duplicated pairs. (**B**) Numbers of genes assigned to each duplication type in the groups shown. Cell labels give gene counts, and color intensity reflects those counts. The Group I column includes the Group I-like member. (**C**) WRKY synteny between *H. brasiliensis* and *Arabidopsis thaliana*. (**D**) WRKY synteny between *H. brasiliensis* and *Manihot esculenta*. Highlighted links indicate WRKY-related collinear pairs, and background links show genome-wide collinearity. The chromosome labels in the cassava synteny panel (**D**) correspond to the original sequence identifiers in the reference genome annotation and were retained to ensure data consistency and traceability.

*HbWRKY* duplication links were distributed across all 18 rubber tree chromosomes rather than being confined to one region (Figure 2A). Of the 109 genes, 102 (93.6%) were assigned to whole-genome or segmental duplication, five (4.6%) to dispersed duplication, and two (1.8%) to proximal duplication (Figure 2B; Table S1). The whole-genome/segmental class comprised the atypical gene, the Group I-like gene, 17 Group I, 70 Group II, and 13 Group III genes. Four Group I genes and one Group II gene were dispersed duplicates; one Group II and one Group III gene were proximal duplicates.

The interspecific analysis detected 130 collinear pairs between *H. brasiliensis* and *A. thaliana*, involving 81 *HbWRKY* and 53 *Arabidopsis* genes, and 302 pairs between *H. brasiliensis* and *M. esculenta*, involving 107 *HbWRKY* and 99 cassava genes (Figure 2C,D). The rubber tree-cassava panel contained a denser set of *HbWRKY*-related links than the rubber tree-*Arabidopsis* panel.

*Ka*/*Ks* was calculated for 111 duplicated *HbWRKY* pairs (Table S3). *Ka* ranged from 0.0384 to 0.5909, *Ks* from 0.1589 to 4.8014, and *Ka*/*Ks* from 0.0478 to 0.5839. All 111 duplicated *HbWRKY* gene pairs exhibited *Ka*/*Ks* ratios below 1, consistent with purifying selection. Among the rubber tree-cassava pairs, 290 pairs involving 106 *HbWRKY* and 99 cassava genes yielded retained *Ka*/*Ks* estimates; all ratios were below 1 (Table S4).

**Figure 3:**
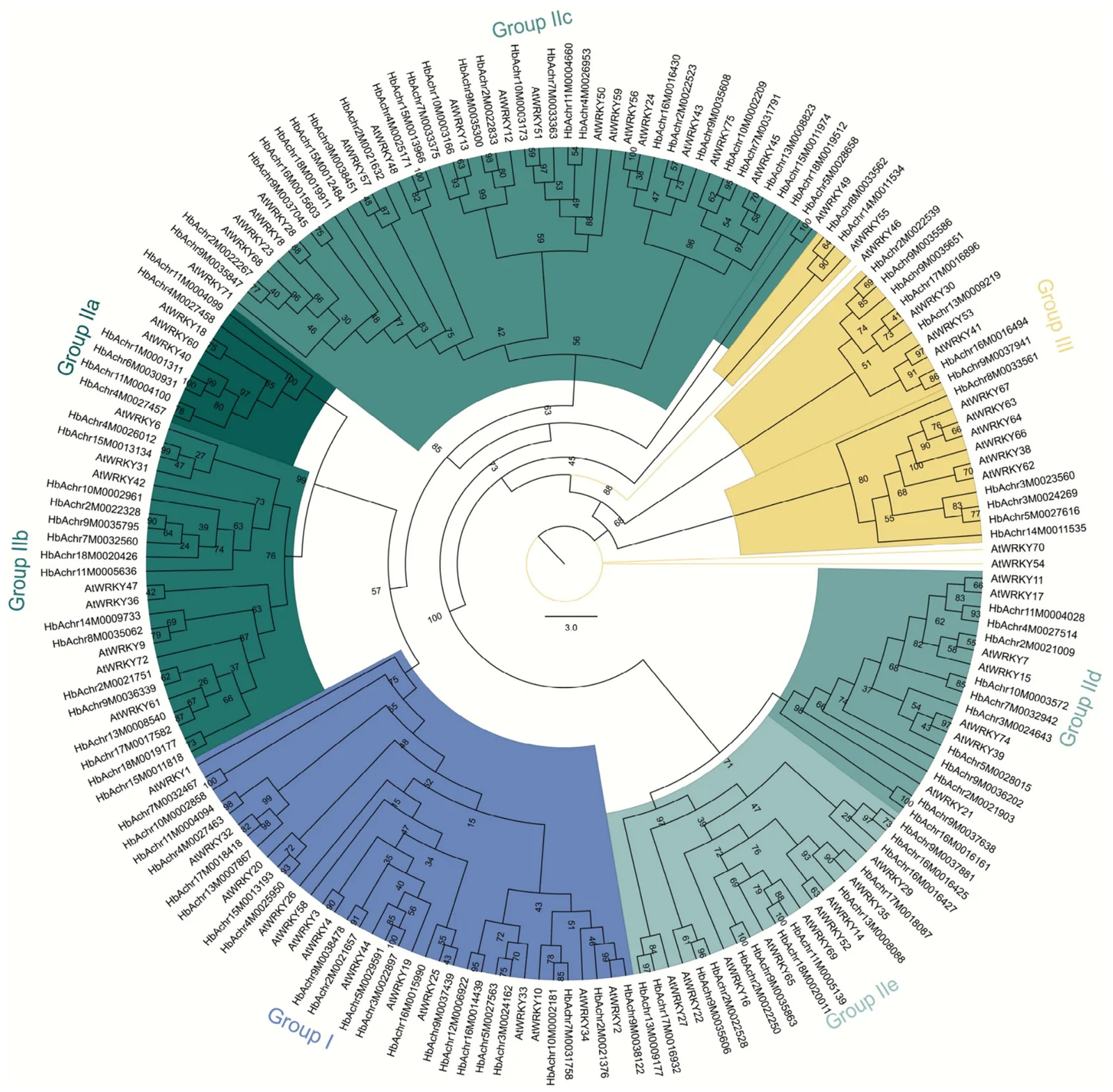
Maximum-likelihood phylogeny of WRKY proteins from *Hevea brasiliensis* and *Arabidopsis thaliana*. Proteins were classified as Group I, Group IIa–IIe, or Group III according to phylogenetic placement and WRKY-domain characteristics. Background colors identify groups and subgroups, and internal-node values show branch support from 5000 ultrafast bootstrap replicates.

The combined rubber tree-*Arabidopsis* phylogeny resolved Group I, Group III, and the five Group II subgroups IIa–IIe (Figure 3). HbWRKY proteins were interspersed with AtWRKY proteins rather than forming a rubber tree-specific cluster. For example, HbAchr10M0002858 and HbAchr7M0032467 clustered with AtWRKY1, HbAchr3M0024162 and HbAchr5M0027563 clustered with AtWRKY33, and HbAchr10M0002181 and HbAchr7M0031758 were positioned near AtWRKY10 (Table S5). The complete assignment table contained 181 phylogenetic-proximity links between 87 HbWRKY and 72 AtWRKY entries.

### 3.3. Tissue Expression and Brassinosteroid Responses

**Figure 4:**
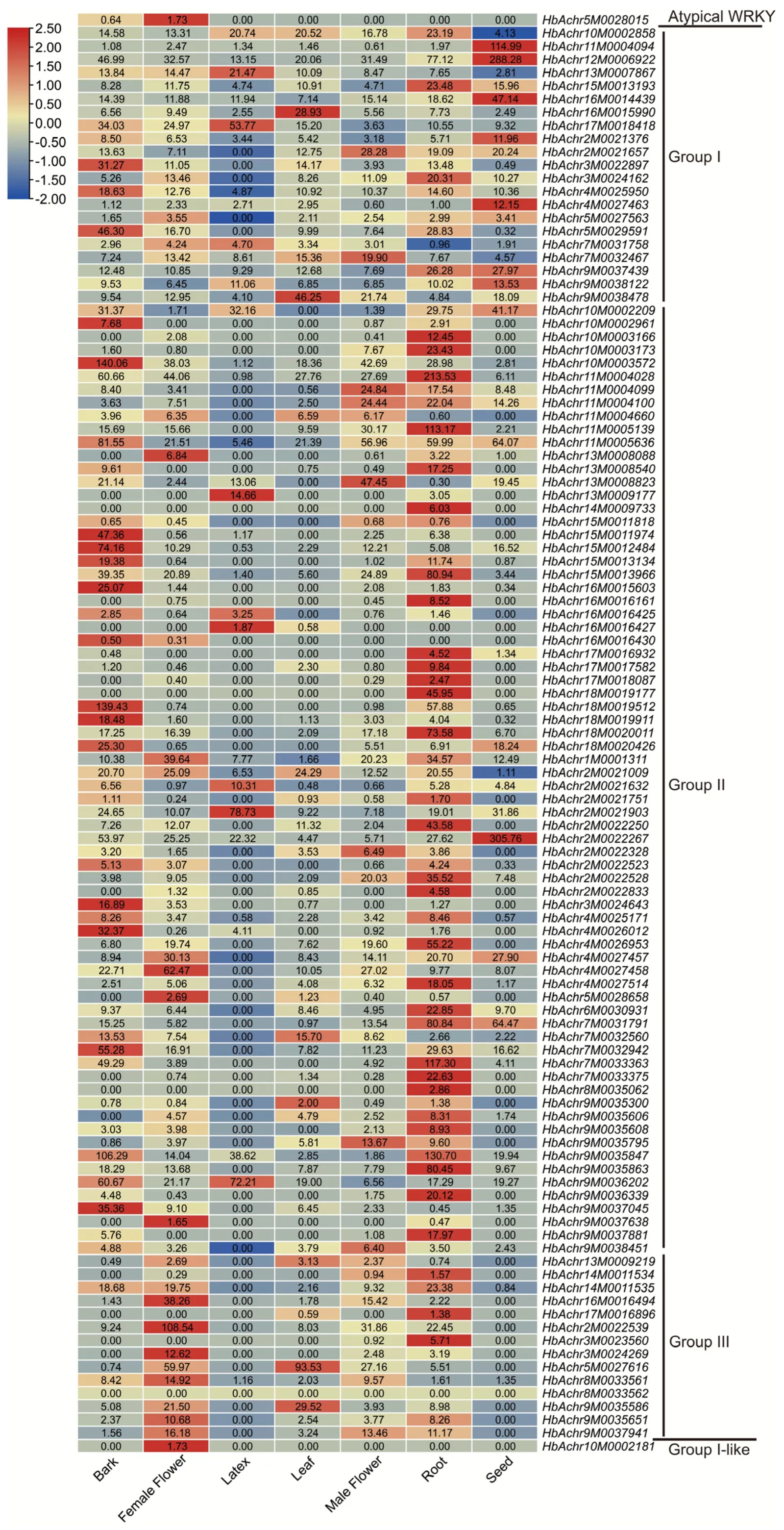
Tissue−expression profiles of *HbWRKY* genes in *Hevea brasiliensis*. Values were standardized by row for visualization; red and blue indicate relatively high and low expression, respectively, for the same gene across tissues. Cell labels show the unscaled matrix values. Genes are arranged by WRKY group.

The tissue-expression heatmap showed broad and tissue-biased patterns across bark, female flower, latex, leaf, male flower, root, and seed (Figure 4; hierarchically clustered profiles are shown in Figure S3). Thirty *HbWRKY* genes had nonzero values in all seven tissues. Bark expression was detected for 89 genes, whereas latex expression was detected for 37. Among the seven analyzed sample types, nine *HbWRKY* genes exhibited their highest recorded transcript abundance in latex samples, including Group I members *HbAchr13M0007867* and *HbAchr17M0018418*, Group IIe member *HbAchr13M0009177*, Group IIc member *HbAchr2M0021632*, and Group IId members *HbAchr2M0021903* and *HbAchr9M0036202*. Nineteen genes showed their highest value in bark. The five largest bark values were recorded for Group IId *HbAchr10M0003572*, Group IIc *HbAchr18M0019512* and *HbAchr9M0035847*, Group IIb *HbAchr11M0005636*, and Group IIc *HbWRKY28*/*HbAchr15M0012484*.

**Figure 5:**
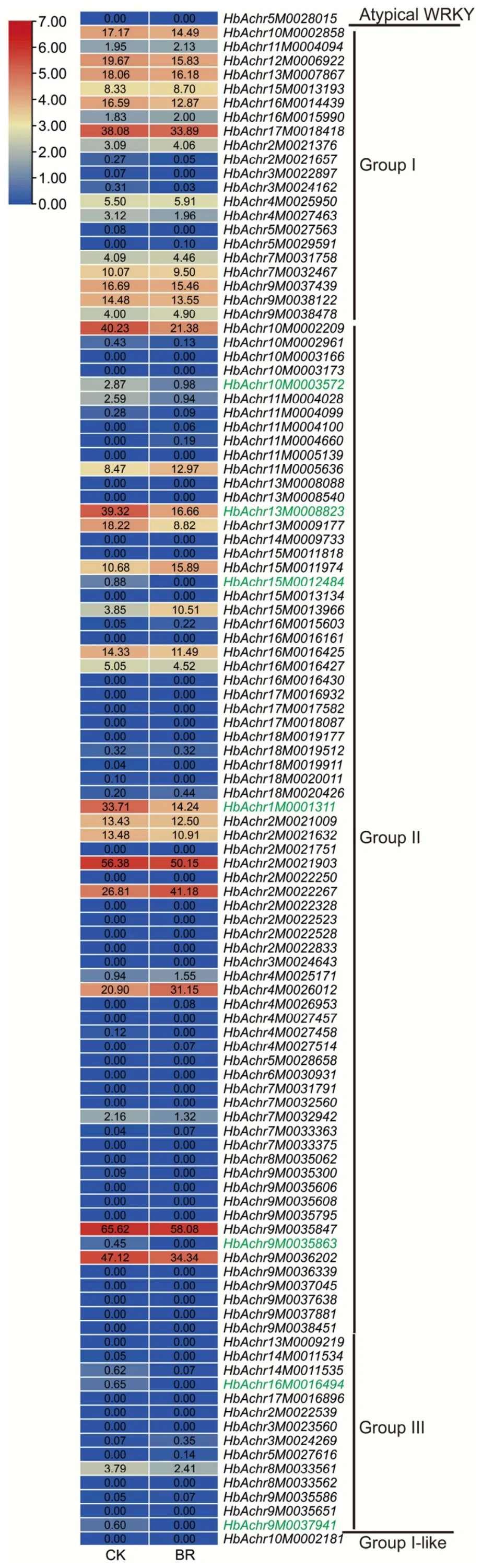
Expression responses of *HbWRKY* genes to brassinosteroid treatment. FPKM values under control (CK) and brassinosteroid (BR) conditions were transformed using log_2_(FPKM + 1) for heatmap visualization. Red and blue indicate high and low log_2_(FPKM + 1) values, respectively, on a common scale across genes. Cell labels show the unscaled condition means. Green gene labels identify differentially expressed genes.

**Figure 6:**
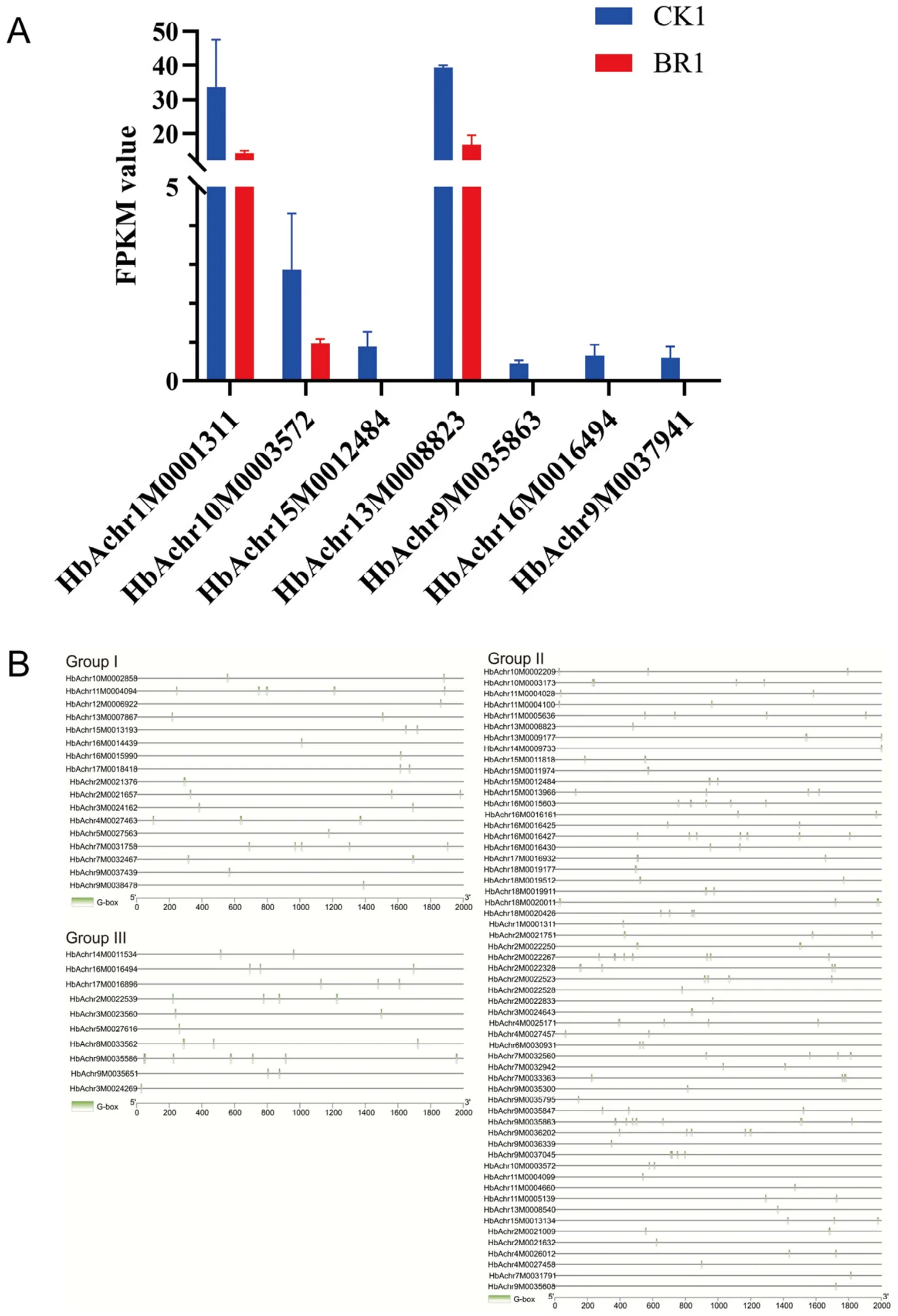
Expression profiles of BR-responsive *HbWRKY* genes and distribution of predicted G-box cis-regulatory elements in *HbWRKY* promoters. (**A**) Expression levels of seven differentially expressed *HbWRKY* genes in latex under control (CK) and brassinosteroid (BR) treatment conditions. Bars represent the mean FPKM values of three RNA-seq libraries per condition, and error bars indicate the standard deviation (SD). The panel labels CK1 and BR1 denote the CK and BR condition means, respectively. (**B**) Distribution of predicted G-box cis-regulatory elements in the promoter regions of *HbWRKY* genes classified into Group I, Group II, and Group III. Horizontal lines represent promoter regions, and green vertical marks indicate the positions of predicted G-box elements. Only promoters with recorded G-box annotations are shown.

Differential expression analysis identified seven *HbWRKY* genes that were significantly downregulated in latex samples collected 12 h after BR treatment compared with the CK samples: *HbAchr1M0001311*, *HbAchr10M0003572*, *HbAchr15M0012484*, *HbAchr13M0008823*, *HbAchr9M0035863*, *HbAchr16M0016494*, and *HbAchr9M0037941* (Figure 5 and Figure 6A; hierarchically clustered profiles are shown in Figure S4). All seven had lower transcript abundance under BR treatment than under CK. *HbAchr1M0001311* decreased from 33.71 to 14.24, *HbAchr13M0008823* from 39.32 to 16.66, and *HbAchr10M0003572* from 2.87 to 0.98. The other four genes had low CK values and were undetected after BR treatment; *HbAchr9M0035863* decreased from 0.45 to 0.

### 3.4. Integrated Prioritization of Brassinosteroid-Responsive Candidates

After exact duplicate coordinates were collapsed, 84 of 109 *HbWRKY* promoters (77.1%) contained G-box elements, yielding 255 unique intervals (Figure 6B; Table S6). G-box-containing promoters occurred in 17 of 21 Group I, 57 of 72 Group II, and 10 of 14 Group III genes; none were recorded for the atypical or Group I-like gene. Unique intervals spanned coordinates 24-2004, consistent with the approximately 2 kb promoter definition. Groups I, II, and III contained 35, 155, and 29 unique sites, respectively.

Five BR-responsive genes belonged to Group II: *HbAchr1M0001311* (IIa), *HbAchr10M0003572* (IId), *HbAchr15M0012484* and *HbAchr13M0008823* (IIc), and *HbAchr9M0035863* (IIe). *HbAchr16M0016494* and *HbAchr9M0037941* belonged to Group III. All seven were assigned to whole-genome or segmental duplication. Six promoters contained at least one G-box. *HbAchr9M0035863* contained eight unique intervals; *HbAchr15M0012484* and *HbAchr16M0016494* contained three each; *HbAchr10M0003572* contained two; and *HbAchr1M0001311* and *HbAchr13M0008823* contained one each. No G-box was recorded for *HbAchr9M0037941*.

*HbAchr10M0003572* and *HbAchr15M0012484* provided the strongest combined evidence for a bark-associated response: Among the seven analyzed tissues, *HbWRKY7* and *HbWRKY28* exhibited their highest recorded transcript abundance in bark, with expression values of 140.06 and 74.16, respectively. Both genes also contained G-box elements in their promoter regions. *HbAchr13M0008823* and *HbAchr1M0001311* were secondary candidates because each was expressed in bark and latex, downregulated following BR treatment, and associated with a G-box. *HbAchr9M0035863* was retained as another secondary bark-associated candidate because it was expressed in bark (18.29) and contained eight G-boxes, although it was undetected in latex and its highest expression occurred in root. *HbAchr16M0016494* and *HbAchr9M0037941* had low bark expression and were undetected in latex; *HbAchr9M0037941* also lacked a G-box. Swiss-Prot homology annotations designated *HbAchr10M0003572* and *HbAchr15M0012484* as *HbWRKY7* and *HbWRKY28*, respectively. These annotation-based aliases do not establish one-to-one orthology with *Arabidopsis* proteins.

## 4. Discussion

The haplotype A telomere-to-telomere reference genome yielded 109 HbWRKY members, 28 more than the 81 reported from an earlier assembly [21]. The expanded members retained the established angiosperm WRKY framework: Group I proteins generally contain two WRKY domains, whereas Groups II and III usually contain one and are further distinguished by zinc-finger type and phylogenetic position [15,16]. Concordance among gene structure, motif composition, and phylogenetic grouping indicates that the principal HbWRKY subfamilies remain structurally constrained. The variant WRKYGQK cores, atypical protein, and Group I-like member nevertheless point to localized amino-acid substitutions or domain degeneration that may affect DNA-binding preference, stability, or protein interactions.

Whole-genome or segmental duplication accounted for 102 genes (93.6%), making it the dominant retained duplication class. Plant genome duplications can preserve transcription factors and other network components through dosage balance, subfunctionalization, or neofunctionalization [34]. Ancient polyploidization in a common euphorb ancestor and extensive retained duplication in the rubber tree lineage provide an appropriate genomic context for the *HbWRKY* pattern [4,35]. All 111 duplicated *HbWRKY* gene pairs exhibited *Ka*/*Ks* ratios below 1, suggesting that purifying selection has contributed to the evolutionary conservation of these duplicated genes [30]. Although synonymous substitution rates (*Ks*) have been widely used to estimate the divergence times of duplicated gene pairs based on molecular-clock assumptions [36], such estimates require an appropriate substitution rate and careful consideration of synonymous substitution saturation. Given the wide variation in *Ks* values among duplicated *HbWRKY* gene pairs, absolute divergence times were not estimated in the present study to avoid potentially unreliable evolutionary interpretations. Furthermore, all 290 WRKY gene pairs with valid *Ka*/*Ks* estimates between *H. brasiliensis* and *M. esculenta* exhibited ratios below 1, suggesting that purifying selection has contributed to the conservation of WRKY protein-coding sequences across these two species. This pattern indicates evolutionary constraints on WRKY genes following the divergence of the two lineages, although their functional conservation requires further experimental validation. Denser WRKY synteny with cassava than with *Arabidopsis* is also consistent with the closer relationship and shared duplication history of the two euphorbs. These observations describe evolutionary conservation and retention; they do not by themselves establish functional equivalence or post-duplication specialization.

G-boxes were widespread in the *HbWRKY* family: 84 promoters contained 255 nonredundant sites. In *Arabidopsis*, activated BZR1 and BES1 recognize BRRE and E-box-related elements, including the G-box, and BES1 can cooperate with bHLH factors such as BIM1 [37,38]. BES1 also controls numerous transcription-factor genes, thereby generating layered transcriptional responses [39]. The presence of G-boxes in *HbWRKY* promoters is therefore compatible with some family members acting downstream of, or in conjunction with, BR-responsive transcriptional networks.

Group II contained 155 of the 219 sites, but it was also the largest group. The proportions of G-box-containing promoters were similar across Group I (81.0%), Group II (79.2%), and Group III (71.4%), which argues against inferring subgroup enrichment from raw site counts alone. BZR1 can also cooperate with the G-box-binding factor PIF4 to integrate BR, light, and temperature signals [40], underscoring that G-boxes are not specific to BR. Promoter prediction alone cannot demonstrate occupancy by BES1/BZR1 in vivo. ChIP-qPCR, EMSA, dual-luciferase assays, and targeted G-box mutagenesis are needed to test direct binding and regulatory activity.

*HbWRKY7* and *HbWRKY28* combined three independent observations: downregulation following BR treatment, the highest recorded expression in bark among the seven sampled tissues, and one or more predicted promoter G-boxes. HbWRKY7 belongs to subgroup IId and is phylogenetically close to AtWRKY21, AtWRKY39, and AtWRKY74. AtWRKY21 cooperates with OBERON proteins and other IId WRKY members to balance growth and stress-responsive transcription [41], whereas AtWRKY39 contributes to heat tolerance [42]. These relationships raise the testable possibility that HbWRKY7 participates in balancing hormone-responsive growth and stress adaptation in bark, but they do not assign a conserved function.

HbWRKY28 belongs to subgroup IIc and lies near AtWRKY8, AtWRKY23, AtWRKY28, AtWRKY68, and AtWRKY71. These *Arabidopsis* proteins span diverse functions: AtWRKY23 influences local flavonol synthesis and auxin distribution in roots [43]; AtWRKY71 alters auxin homeostasis and shoot branching [44]; AtWRKY8 contributes to salt tolerance [45]; AtWRKY28 restricts ectopic female germline specification [46]; and AtWRKY68 responds to auxin and high temperature [47]. Their functional breadth cautions against transferring a single role to HbWRKY28. A reasonable working hypothesis is that HbWRKY28 participates in hormone and environmental response networks in bark, potentially including BR-auxin crosstalk, but this remains to be tested.

HbAchr1M0001311 (IIa) groups with the ABA-related AtWRKY18/40/60 clade, and HbAchr13M0008823 (IIc) lies near phosphate-responsive AtWRKY45 [48,49]. The Group III genes *HbAchr16M0016494* and *HbAchr9M0037941* showed low bark expression and were undetected in latex; they group with AtWRKY41 and AtWRKY53, which regulate seed dormancy and leaf senescence, respectively [50,51]. These comparisons generate hypotheses rather than functional assignments. Independent qRT-PCR validation was not performed because the corresponding biological materials and sample information were unavailable. The 12 h transcript profiles may include secondary effects and broader transcriptional adjustments; they do not identify direct or early BR targets. Low-expression candidates, including *HbWRKY28*, therefore require particular caution and independent validation. Demonstrating a BR-WRKY contribution to latex-associated biology will require promoter-binding and transcriptional assays, downstream-target analysis, and gene knockdown or overexpression in an appropriate rubber tree system.

## 5. Conclusions

This study updated the rubber tree WRKY family to 109 members and showed that whole-genome or segmental duplication, coupled with pervasive purifying selection, shaped much of its retained repertoire. Integration of tissue expression, BR-responsive transcript profiles, and promoter G-box occurrence prioritized *HbWRKY7* and *HbWRKY28* as core bark-associated candidates. The analysis narrows the set of genes for mechanistic study but does not establish direct regulation by BR signaling components or a causal contribution to natural rubber biosynthesis.

## Data Availability

The original contributions presented in this study are included in the article/Supplementary Materials.

## Supplementary Materials

The following supporting information can be downloaded at https://www.mdpi.com/article/10.3390/biology15211965/s1, Figure S1: Gene structures of *HbWRKY* family members in *Hevea brasiliensis*; Figure S2: Distribution of conserved motifs in HbWRKY proteins; Figure S3: Hierarchical clustering of *HbWRKY* gene expression profiles across different tissues of *Hevea brasiliensis*; Figure S4: Hierarchical clustering of *HbWRKY* gene expression profiles under control and brassinosteroid treatment conditions; Table S1: Sequence-based classification, WRKY-domain features, and duplication types; Table S2: Physicochemical properties and predicted subcellular localization; Table S3: *Ka*/*Ks* analysis of duplicated *HbWRKY* pairs; Table S4: *Ka*/*Ks* analysis of rubber tree-cassava WRKY pairs; Table S5: Phylogeny-based functional annotation using characterized *Arabidopsis* WRKY homologs; Table S6: Distribution of predicted G-box and other cis-regulatory elements in the promoter regions of *HbWRKY* genes in *Hevea brasiliensis*; Table S7: Expression profiles of *HbWRKY* genes under control and brassinosteroid treatment conditions.
